# Methodology for the Positive Voices 2022 Survey of People With HIV Accessing Care in England, Wales, and Scotland: Cross-Sectional Questionnaire Study

**DOI:** 10.2196/58531

**Published:** 2025-01-10

**Authors:** Janey Sewell, Carole Kelly, Adamma Aghaizu, Hannah Kitt, Annegret Pelchen-Matthews, Veronique Martin, Amal Farah, Colette Smith, Alison Brown, Clare Humphreys, Alex Sparrowhawk, Valerie Delpech, Alison Rodger, Fiona Lampe, Meaghan Kall

**Affiliations:** 1 Department for Infection and Population Health University College London London United Kingdom; 2 UK Health Security Agency London United Kingdom; 3 George House Trust Manchester United Kingdom; 4 University College London London United Kingdom; 5 Royal Free London NHS Foundation Trust London United Kingdom

**Keywords:** HIV care, national survey, quality of life, Positive Voices, transgender population, gender-diverse population, health disparities, health services research, living with HIV, HIV stigma, social determinants of health, longitudinal cohort, access to care, welfare services, health policy, preventive care

## Abstract

**Background:**

Due to advances in treatment, HIV is now a chronic condition with near-normal life expectancy. However, people with HIV continue to have a higher burden of mental and physical health conditions and are impacted by wider socioeconomic issues. Positive Voices is a nationally representative series of surveys of people with HIV in the United Kingdom. It monitors the physical, mental, and social health, well-being, and needs of this population so that they can be addressed.

**Objective:**

This paper aimed to describe the methodology, recruitment strategies, and key demographic features of participants recruited for the second national round of Positive Voices (PV2022).

**Methods:**

PV2022 was a national, cross-sectional questionnaire study that included people attending HIV care at 101 of the 178 clinics in the United Kingdom between April 2022 and March 2023. Data from the HIV and AIDS reporting system (HARS), a national surveillance database of people with HIV and attending care that is held at the UK Health Security Agency (UKHSA), was used as a sampling frame. The information collected in PV2022 included demographic and socioeconomic factors, HIV diagnoses and treatment, mental and physical health, health service use and satisfaction, social care and support, met and unmet needs, stigma and discrimination, quality of life, lifestyle factors, and additional challenges experienced due to the COVID-19 pandemic. Data linkage to HARS enabled the extraction of clinical information on antiretroviral therapy (ART), HIV viral load, and CD4 lymphocyte counts. Probabilistic sampling was used to provide a randomly selected, representative sample of people attending HIV care who could be invited to complete a paper or online questionnaire. At the start of 2023, due to under-recruitment mainly due to the impact of the monkeypox (Mpox) outbreak, a separate sequential recruitment strategy was initiated in 14 of the largest clinics to increase participant numbers.

**Results:**

Of the 4622 participants who completed the questionnaire, 3692 were recruited through probabilistic recruitment and 930 through sequential recruitment. The overall response rate (measured as the number of people who completed a questionnaire of those who either accepted or declined) was 50%. Survey respondents represented approximately 1 in 20 people diagnosed with HIV in England, Wales, and Scotland. The median age of participants was 52 years, 3428 of participants were men, 2991 were White, and 1121 were Black.

**Conclusions:**

PV2022 is currently the largest survey of people with HIV in the United Kingdom (as of September 2024). The PV2022 findings will be used to explore the health and well-being of the HIV population and examine associations with demographic, socioeconomic, lifestyle, and other HIV-related factors.

**International Registered Report Identifier (IRRID):**

RR1-10.2196/58531

## Introduction

In the last 2 and a half decades, advances in HIV treatment have greatly reduced the mortality rate among people with HIV [1]. As a result, HIV is now viewed as a chronic condition with near-normal life expectancy [2]. As the population of people with HIV is ageing (half are now aged 50 years and older) [3] and health service delivery models are undergoing continuous change [4], it is essential to understand the types and levels of health needs of people with HIV and to assess long-term outcomes and quality of life [5].

In 2022, there were 94,397 people diagnosed with HIV and accessing care in England [6]. It was estimated that 95% of people with HIV were diagnosed; 98% of those diagnosed were on treatment; and 98% of those on treatment were virally suppressed and therefore unable to transmit HIV [7,8]. Research has indicated that despite substantial improvements in prognosis for people with HIV on treatment and the high coverage of specialist HIV care, there remains a significant level of psychosocial need among people with HIV [9]. They experience higher levels of mental health symptoms [9], with the prevalence of depression and anxiety around twice as much as the general population [10-12]. High levels of perceived and internalized stigma associated with HIV status are experienced by people with HIV, impacting their mental health [13]. Socioeconomic issues, such as poverty, unemployment, unstable housing, lack of social support, and intimate partner violence have also been shown to impact people with HIV, affecting both physical and mental health [14-16]. Understanding these needs is particularly crucial following a period of significant changes in health and social care service provision, due to policies of austerity and the COVID-19 pandemic [17]. Positive Voices (PV) collects and analyses these types of data, providing valuable insights that are used to inform national HIV policy and prevention programs and to evaluate and commission HIV specialist services.

PV is a cross-sectional questionnaire study of people with HIV who receive HIV specialist care in England, Wales, and Scotland, carried out every 3-5 years. In 2014, an extensive pilot phase that included formative research with the HIV community and health care staff facilitated the development of the survey methodology, which was subsequently used for the national roll-out in 2017 (Positive Voices 2017 [PV2017]) [9,18]. Our paper aims to describe the methodology and study design of the second round of the study: Positive Voices 2022 (PV2022).

## Methods

### Study Design

PV2022 was a national, cross-sectional questionnaire survey of people with HIV attending HIV outpatient care in England, Wales, and Scotland. The questionnaire data were linked to clinical data on antiretroviral therapy (ART), HIV viral load, and CD4 lymphocyte count from the HIV and AIDS reporting system (HARS), a national surveillance database of people with HIV attending care, held at the UK Health Security Agency (UKHSA).

### Setting

In December 2020, all HIV clinics that routinely report to HARS were emailed by the PV team and asked whether they wished to express an interest in becoming a PV2022 study site. A total of 101 clinics, over half (57%) of the 178 HIV clinics in the country, returned an expression of interest, 98% of whom were in England (99/101), 1 was in Scotland (1/101), and 1 in Wales (1/101). Sixty-two (61%) clinics had previously participated in the previous round of the study (PV2017). Initially, the recruitment of participants was planned to begin in 2021, but was delayed as patient attendance at clinics was disrupted due to the COVID-19 pandemic restrictions. In April 2022, the recruitment of participants began, as services had started returning to face-to-face consultations. However, following the pandemic, many clinics had adopted a hybrid approach of giving remote telephone or video consultations as well as seeing patients in clinic. Refer to Multimedia Appendix 1 for a list of participating clinics.

In this paper, “clinic staff” has been defined as clinicians (nurses or doctors) and researchers who worked in the participating HIV clinics.

### Sample Size

HARS was used as a sampling frame to provide a representative, random sample of people with HIV within each participating clinic who could be approached to participate in the PV2022 survey. HARS is a surveillance database held at the UKHSA that consists of pseudonymized data on the demographic, clinical, and treatment characteristics of people with HIV that is reported each quarter year by all HIV service providers in England [19,20].

The population of patients attending the participating clinics was approximately 74,000. The sample was to include 1 in 5 patients attending each of these clinics. Data from the pilot study and PV2017 indicated a proportion of individuals would be nonrecruitable due to being deceased, no longer attending clinic, being lost-to-follow-up, having been imprisoned or having an incorrect hospital identification number. Therefore, considering this, a sample list of 17,121 patients was created with the assumption that approximately 14,400 would be recruitable. Based on the response rate in PV2017 and observed declines in response rates in other large population-based studies in recent years, the PV2022 response rate was expected to be between 30% and 50%, resulting in 4320 to 7200 participants being recruited. The precision of prevalence estimates of measures such as diagnosed depression, occurring at approximately 30% prevalence, would therefore be ±1.4% and ±1.1% (with 95% confidence) for the response rates of 30% and 50%, respectively. Within a key demographic subgroup comprising 10% of the population, the precision would be ±4.4% and ±3.3%, respectively.

### Study Population and Selection

Clinic staff were asked to approach their patients who had been randomly sampled from HARS and included in a list provided by the PV team. Each selected patient was assigned a unique identifier that was displayed on their survey packs or could be used to access their online questionnaire.

The PV team had made efforts to oversample the transgender and gender-diverse HIV population by adding to each clinic’s list all patients recorded on HARS who identified as trans or gender-diverse. This was carried out to ensure a sufficient number of participants from this population group were represented, as it had been reported that this cohort was experiencing larger health inequalities in comparison with the general population [8].

### Inclusion Criteria

The inclusion criteria were people diagnosed with HIV, aged 18 years or older, residing in the United Kingdom, accessing care at a participating HIV clinic in England, Wales, or Scotland, and able to complete the questionnaire in English either online or on paper.

### Exclusion Criteria

There were no set exclusion criteria, however the preselected patients were defined as nonrecruitable if they were unable to take part in the survey for any of the following reasons: moved abroad, transferred care to another clinic, lost contact with the clinic for more than 12 months, had died, had any mental or emotional issue affecting their ability to give implied consent, had a language barrier or literacy difficulties, or were imprisoned or deemed a vulnerable adult.

### Study Management

PV2022 was a collaboration between the University College London (UCL) NICHE team, the UKHSA HIV national surveillance team, and a NICHE Patient and Public Involvement representative. “A Person-Centered Needs Informed Model of Care for People with HIV” (NICHE) is a National Institute for Health Research (NIHR) funded program of research to improve the health and well-being of people with HIV. The PV team oversaw all aspects of the research including the study design, the development of the questionnaire and study documents, the oversight of the ethics and governance processes, the monitoring of response rates and recruitment at the participating sites and considered strategies to support and boost recruitment.

### Questionnaire Development

Half of the questions in PV2022 were identical to those in PV2017 and were considered core questions to the survey series. These included demographic and socioeconomic factors, HIV related factors, comorbidities, met and unmet health, social and welfare service needs, quality of life measured by EQ-5D-5L, height and weight, smoking and alcohol status by the Alcohol Use Disorders Identification Test-Consumption (AUDIT-C), recreational drug use and general practice (GP), and HIV clinic satisfaction, enabling assessment of trends over time in these factors. New items included in the PV2022 questionnaire included self-reported HIV viral load (whether undetectable or not at last test); questions exploring how well participants understood and trusted the concept of Undetectable equals Untransmittable (U=U), if U=U knowledge and trust impacted on how they felt about their HIV status, a modified version of the Duke-UNC (University of North Carolina) Functional Social Support Questionnaire [21], the 14-item Resilience Scale (RS14) [22], the General Practice Physical Activity Questionnaire (GPPAQ) [23], and questions on the impact of COVID-19 and the uptake of COVID-19 vaccines. In addition, the measure for anxiety and depression in 2017 [24] was replaced with standardized separate measures for depression and anxiety symptoms: the Patient Health Questionnaire-9 (PHQ-9) [25], and the General Anxiety Disorder Questionnaire-7 (GAD-7) [26]. Questions on stigma and discrimination were expanded to take account of internalized and other stigma and included an adapted validated stigma scale [27]. In addition, participants who completed the study online in PV2022 were able to opt-in to complete “Positive Outcomes,” an HIV Patient Reported Outcome Measure (PO-PROM), a tool designed for use in clinical settings to assess the needs and concerns of people with HIV [28]. Table 1 details the topics included in PV2017 and PV2022 questionnaires. The PV2022 questionnaire was evaluated online by two groups of people with HIV (up to 10 per group) during November and December 2021. Members of these groups had previously participated in PV2017 and had provided their consent to be contacted again to take part in further research. They provided helpful feedback on the length of the questionnaire, the readability and phrasing of questions, and the flow and overall order of questions. The questionnaire was modified accordingly.

**Table 1:** Questionnaire topics, questions, survey tools, and clinical data collected in Positive Voices 2017 and Positive Voices 2022.

Questionnaire topic		PV2017^a^	PV2022^b^
Demographics (age, gender, ethnicity, and sexual orientation)		✓	✓
Socioeconomics (education, employment, housing, financial status, and religion)		✓	✓
HIV diagnosis and treatment (year and country of diagnosis, year started on ART^c^, adherence, and side effects)		✓	✓
Self-reported HIV viral load and when last test conducted		—^d^	✓
U=U^e^ (understanding of and trust in U=U)		—	✓
Non-HIV diagnosed conditions and treatments (cardiovascular conditions, joint and bone, cancer, mental health, and other long-term conditions)		✓	✓
Mental health and well-being (mental health symptoms [General Health Questionnaire, GHQ-12])		✓	—
Mental health and well-being (depression symptoms [Patient Health Questionnaire, PHQ-9], anxiety symptoms [Generalized Anxiety Disorder questionnaire, GAD-7], modified DUKE-UNC Functional Social Support questionnaire [DUKE-FSSQ], and the Resilience Scale [RS14])		—	✓
Quality of life, life satisfaction and self-rated health (EQ-5D-5L,Office for National Statistics Life Satisfaction and self-rated health questions)		✓	✓
Disclosure and discrimination (disclosure, discrimination in health care settings)		✓	✓
Stigma (internalized stigma and anticipated stigma)		—	✓
Sex and relationships (partner status, sexual behavior, sexually transmitted infections diagnoses)		✓	✓
Women’s sexual and reproductive health (pregnancy and contraception)		✓	—
Sexual satisfaction (physical and emotional sexual satisfaction)		—	✓
General health and lifestyle (height, weight, smoking, alcohol [Alcohol Use Disorders Identification Test-Consumption, AUDIT-C] and recreational drug use)		✓	✓
General health and lifestyle (exercise and physical activity [General Practice Physical Activity Questionnaire, GPPAQ])		—	✓
GP^f^ and HIV service use and satisfaction (GP and HIV satisfaction, patient experience measures, and use of GP and HIV support services)		✓	✓
Health service use and satisfaction (expanded list of health and social care service use)		—	✓
Impact of COVID-19 pandemic (COVID-19 diagnosis, vaccination status, and access to technology for online appointments)		—	✓
Met and unmet needs (need for HIV related services, health services, social and welfare services)		✓	✓
HIV Positive Outcomes Patient Reported Outcome Measure (PO-PROM^g^) (online questionnaire only, opt-in option)		—	✓
Clinical information from HARS^h^			
	Antiretroviral treatment regimen	✓	✓
	Laboratory measures (viral load and CD4 lymphocyte count)	✓	✓

^a^PV2017: Positive Voices 2017.

^b^PV2022: Positive Voices 2022.

^c^ART: antiretroviral therapy.

^d^—: not applicable.

^e^U=U: Undetectable=Untransmittable.

^f^GP: general practitioner.

^g^PO-PROM: Positive Outcomes patient-reported outcome measure.

^h^HARS: HIV and AIDS reporting system.

### Study Documents

Each participating clinic was provided with electronic copies of the study materials including the protocol, manual of operations, participant information leaflet, privacy notice, waiting room poster, and a password-protected study log that listed each clinic’s sample list of randomly selected participants. Study documents such as the protocol were also available on the study website [29]. Questionnaire packs, 1 for each participant within the clinic’s sample, were dispatched by courier to the site. The pack consisted of a sealed envelope labelled with the participant’s clinic number, hospital name, and unique identifier displayed on the outside, as well as the patient information leaflet, questionnaire, a freepost envelope, and a signposting sheet detailing community support organizations such as the Terrence Higgins Trust, a British charity that campaigns and provides services relating to HIV and sexual health.

### Site Initiation Meetings

Each participating site attended 1 of the site initiation meetings that took place remotely on Microsoft Teams between February and March 2022. A maximum of ten clinic sites attended each meeting. Information on how to identify, contact, and recruit participants was explained, and a demonstration was given on the procedure for completing the study log (refer to Recruitment and Data Collection section). Sites were asked to nominate a PV2022 champion within their clinic who would act as the main contact with the PV team. All the site initiation meetings were completed by March 18, 2022, just before the recruitment of participants commenced in April 2022.

### Recruitment and Data Collection

The first participant was recruited on April 11, 2022, and recruitment closed on March 31, 2023. At each site, clinic staff were asked to complete a study log on their recruitment outcomes and return it to the PV team each month. The information they recorded in their study log included the mode (whether in-person, email, text, or post) and date of each participant’s initial contact, the survey distribution method (in-person, email, text, or post), the survey distribution date, the recruitment status (whether nonrecruitable, accepted, or declined), and the reason if the status was nonrecruitable.

The PV team provided a brief script for email and text message approaches as a guide for clinic staff. Online questionnaires were sent by a link by email or text message, or patients received their survey pack containing the paper questionnaire at their next clinic attendance. Clinic staff decided on the method of approach used in accordance with each participant’s preferred and usual method of communication with their clinics.

### Change to the Sequential Recruitment Strategy at Specific London Sites

Reviewing the study logs monthly, it was established that several sites were underrecruiting. Consequently, the recruitment period was extended from 6 to 12 months to allow clinics additional time to reach their recruitment targets. Further email correspondence with clinics revealed that many had resource pressures, fewer face-to-face clinic appointments after the start of the COVID-19 pandemic, and the 2022 Mpox outbreak had been an unanticipated challenge impacting upon recruitment, particularly in London. In response, the PV team designed a sequential recruitment strategy whereby a site could approach any eligible participant attending the clinic sequentially, that is, approach patients as they entered the clinic instead of contacting participants from their preselected sample list. In total, 14 HIV clinics across 7 NHS trusts in London that had recruited less than 10% of their minimum target after 8 months were offered a switch to the sequential recruitment strategy, which they commenced at the beginning of December 2022, having been sent new recruitment packs, survey labels, and a simpler study log that reflected the new strategy.

Participants recruited through the new sequential recruitment strategy were only able to complete the paper questionnaire. Staff were asked to encourage participants to complete their questionnaires in the clinic when they came for their consultation rather than taking them home, as questionnaires sometimes got lost, forgotten, or were not returned to the PV team at UKHSA.

### Secure Storage and Transfer of Data

Paper questionnaires and study logs were securely stored within each clinic. The sites were responsible for collecting, storing, and returning the paper questionnaires completed in their clinic to the PV team at the UKHSA by using the Freepost envelopes provided in the participant packs or by courier.

### Data Management

When paper questionnaires were received by the PV team, data were double data-entered (entered by 2 separate data input personnel) into Snap Survey software (Snap Survey Ltd) and each questionnaire was scanned. Online responses were extracted from Snap Survey as an electronic csv file. The data from both sources was then imported into Stata (version 15.0, StataCorp) and merged to create a master questionnaire data file. Data discrepancies between the 1st and 2nd date entries of the paper questionnaires were checked and resolved by referring to scanned copies of questionnaires.

### Linkage

Data on ART use, the most recent CD4 lymphocyte count, and HIV viral load measurement were linked from HARS to the survey data using the participant’s clinic ID number and unique identifier.

### Data Security

Paper questionnaires were stored securely in locked cabinets at UKHSA where they will remain for a maximum of 10 years and then be securely destroyed. The online version of the questionnaire was hosted on a secure HTTPS connection and data were encrypted at the point of transmission and stored on a secure, dedicated, virtual server hosted at UKHSA.

All data were securely held at UKHSA, encrypted, and restricted to the PV team. Data collection, storage, and use were consistent with the procedures described in the NHS Information Governance Toolkit. A data-sharing agreement was developed to ensure the secure transfer of pseudonymized data between UKHSA and UCL for data analysis.

All researchers were trained to handle data according to Caldicott guidelines, the General Data Protection Regulation, and Section 60 of the Health and Social Care Act.

### Ethical Considerations

The Positive Voices study was granted ethical approval by the London Harrow Research Ethics Committee (13/LO/0279) on March 28, 2013. All the updated documents for PV2022 were submitted as a substantial amendment and subsequently approved on June 23, 2021. The patient information leaflet provided information on how the patients could participate, the potential risks and benefits of participation, and contact information for the PV team. Clinic staff were advised to tell each eligible participant that participation was voluntary and that their decision to participate would not affect their care. A privacy notice was available online with further information about data security [29]. Consent was implied on voluntary completion of the questionnaire. All participants received a digital gift voucher for 5 GBP (equivalent to US $6.50) as an acknowledgement for their time and consideration. This incentive was chosen based on findings from the formative work undertaken before the survey began, where participants expressed a requirement for an incentive. The PV team provided monthly feedback to sites showing the number of completed questionnaires received either by post or online from participants from their clinic. If a patient agreed to participate in the survey but no questionnaire had been returned, clinic staff were asked to send up to 3 reminders using the preferred method of contact initially used. Participants were not asked to provide identifiable information for the survey. However, at the end of the survey, participants were given the option of providing their personal contact details, either phone number or email address, if they were willing to be contacted for future research. If contact details were given, this personal information was unlinked from their survey data and stored securely on a separate password-protected database at UKHSA.

## Results

### Overview

The PV2022 survey was completed by 4622 people diagnosed with HIV, representing approximately one in 20 people living with HIV and accessing care in England, Wales, and Scotland in 2022 [19]. Overall, 4540 participants provided sufficient demographic information to enable linkage to HARS to acquire additional clinical data, which included treatment information, viral load, and CD4 lymphocyte count. The most popular completion method was by paper questionnaires with 2829 (61%) compared with 1793 (39%) online questionnaires. A third (930/2829, 33%) of the paper questionnaires were completed by participants recruited sequentially. Of the 4622 participants, 1737 provided their phone numbers or email addresses as contact details regarding further research, and 897 completed the optional PO PROM questionnaire online.

### Response Rate

To calculate the response rate, the number of completed questionnaires (n=4622) was divided by the number of people who accepted (n=8096) or declined to participate (n=1088), which gave an overall response rate of 50%. In addition, 4994 people had been contacted by email or text but had not responded, which may have reflected further declines although may also have been due to messages not being received, read, or being discounted as an unsolicited message. By including these nonresponses in the calculations, it would have given a response rate of 33%. The true response rate likely lies somewhere between 33% and 50%.

**Figure 1:**
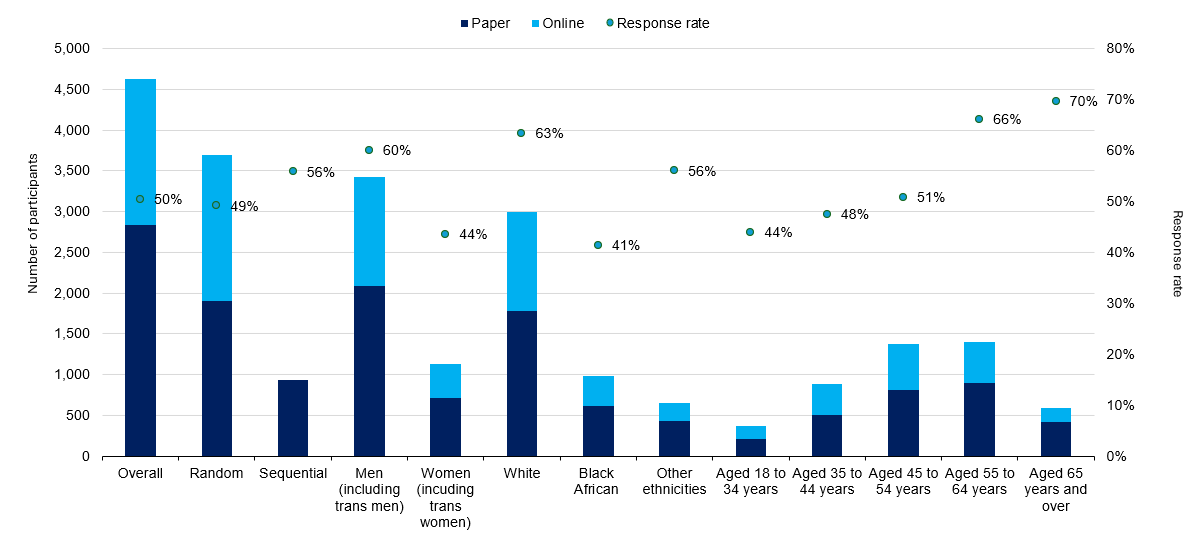
Positive Voices 2022 response rates by recruitment mode, gender, ethnicity, and age among participants of Positive Voices 2022 (N=4622).

A total of 930 (20%) participants were recruited through the sequential recruitment strategy, a higher response rate (55%) compared with the original preselected participant recruitment strategy (49%, Figure 1). Of the 930 sequentially recruited participants, 906 were able to be matched to HARS: 222 (25%) of these had initially been preselected to take part in the survey. Response rates differed by demographic characteristics (taken from HARS) as shown in Figure 1. Men had a higher response rate (60%) (3428 completed out of 5715 accepted or declined) compared with women (1124/2580, 44%) and White people had a higher response rate (2991/4724, 63%) compared with Black African people (983/2380, 41%) or those of other ethnicities (648/1158, 56%). Response rates were also higher in older age groups, with the highest response rate (592/851, 70%) in the people aged 65 years or older (Figure 1). Multimedia Appendix 2 provides the corresponding numbers for all groups shown in Figure 1.

### Age, Gender, and Ethnic Distribution of PV2022 Participants

The median age of PV2022 participants (n=4620) was 52 (IQR 43-60) years, and about 3 quarters of participants had been diagnosed with HIV more than 10 years ago (Table 2). Nearly a quarter of participants were women (24%) and over a fifth (21%) of participants were Black African people (Table 2).

**Figure 2:**
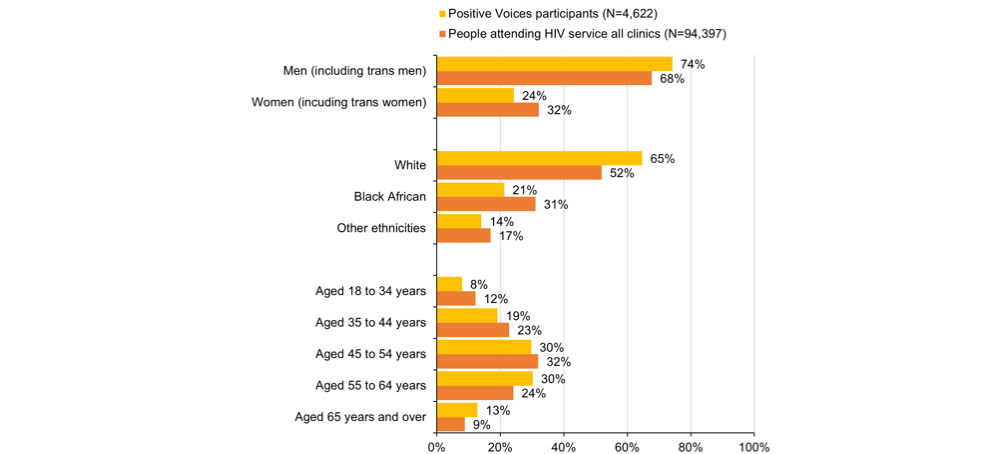
Representativeness of Positive Voices 2022 participants compared with the national population of people with HIV who attended HIV services in 2022.

Gender identity of participants completing PV2022 broadly reflected the gender identity distribution of people accessing HIV care in the United Kingdom. However, there was some overrepresentation of men (6% higher), and underrepresentation of women (8% lower) compared with HARS data (Figure 2). A total of 67 PV2022 participants identified as transgender or gender diverse. 

Compared with the national population of people accessing HIV care, Black African individuals were underrepresented in the PV2022 sample (10% lower), and White participants were overrepresented (13% higher; Figure 2). 

Participants from the sequential recruitment route were younger compared to the preselected random recruitment strategy (median age 49 (IQR 39-57) years versus 53 (IQR 45-60) years, for sequential versus random recruitment respectively), overrepresented men (85% vs 72%) and White people (67% vs 64%) and underrepresented Black African people (14% vs 23%).

A higher proportion of participants who completed the online questionnaire were White compared to participants who completed the paper questionnaire (67% online vs 63% paper; Figure 1 and Multimedia Appendix 2.

**Table 2:** Demographics and year of HIV diagnosis among Positive Voices 2022 participants.

Characteristics		Participants (n=4622), n (%)
Age group (years)^a^		
	18-34	369 (8)
	35-44	885 (19.2)
	45-54	1375 (29.8)
	55-64	1397 (30.3)
	65+	592 (12.8)
Gender		
	Man (including trans men)	3428 (74.2)
	Woman (including trans woman)	1124 (24.3)
	Nonbinary	26 (0.6)
	In another way	8 (0.2)
	Prefer not to say and unknown	36 (0.8)
Ethnic group		
	White	2991 (64.7)
	Black African	983 (21.3)
	Black other	138 (3)
	Asian	189 (4.1)
	Mixed	143 (3.1)
	Other (including prefer not to say and unknown)	178 (3.9)
Year of diagnosis		
	2019-2023	214 (4.6)
	2014-2018	801 (17.3)
	2009-2013	957 (20.7)
	2004-2008	1063 (23)
	2003 or earlier	1497 (32.4)
	Unknown	90 (1.9)

^a^Age was missing for 2 participants.

### Survey Distribution Method

The survey approach method was available for 4375 of the 4622 responses received. Over half of these participants, 54% (2371/4375), were approached in person, 23% (1001/4375) were contacted by phone, 17% (727/4375) received a text message, 5% (214/4375) were contacted by email, and 1% (62/4375) received the questionnaire by post.

## Discussion

### Principal Findings

In this paper, we have described the methods, design and sample demographic characteristics of the Positive Voices 2022 study. PV2022 is the largest questionnaire study to date of people with HIV in England, Wales, and Scotland. The outputs from the study will provide a unique insight into the health and well-being of people with HIV, particularly regarding mental health, met and unmet needs, experiences of stigma, and the impact of COVID-19 pandemic [30]. The overall response rate for PV2022 was estimated to be between 33% and 50% compared with 52% for the previous iteration of the survey (PV2017) [9]. Several factors impacted on the response rate in PV2022. In particular, the first 6 months of the recruitment period (between April and September 2022) coincided with the Mpox *clade* IIb outbreak, primarily among gay and bisexual men in London and other large cities in England [31]. The impact of this outbreak on sexual health and HIV services resulted in a significant deployment of clinic staff and resources to manage the outbreak and to provide a vaccination program to contain the infection [31]. Feedback from some of the largest clinics that were open to recruitment for PV2022 over the duration of the Mpox outbreak suggested that recruitment had been challenging to prioritize during this period, particularly as the methodology required considerable clinic staff time commitment.

As a result of a challenging recruitment environment, the switch to sequential recruitment in December 2022 resulted in an improvement to study recruitment. This was mainly because the strategy of sequential recruitment did not require the administration time that had been required for the original strategy. Furthermore, as these participants were only able to complete the paper questionnaire and not the online version, clinic staff recommended questionnaires be completed within the clinic setting and discouraged participants from taking the questionnaires home, where they may have remained uncompleted or unreturned. Although this strategy was not always successful, the proportion that completed and submitted their questionnaire within the clinic was higher in comparison with the original recruitment strategy.

Other potential factors that may have impacted upon the response rate for PV2022 related to the COVID-19 pandemic. The widespread adoption of online health care appointments during the pandemic meant that fewer patients attended regular HIV appointments in person [32,33]. A recent systematic review identified interruption in attendance of in-person clinic consultations, reduced adherence to treatment, and a subsequent increase in mortality among people with HIV, as a consequence of the changes in service delivery due to the COVID-19 pandemic [34]. As the most effective mode of recruitment for PV2022 was in-person with clinic staff, the decreased frequency of participants attending clinic likely contributed to the lower response rate seen in PV2022 in comparison with PV2017. Less personal interaction between patients and clinic staff may also have impacted upon participants’ enthusiasm for completing questionnaires when approached. When clinic staff were able to personally hand out the survey packs within the clinic, they were better able to explain the importance of the study and the impact the results would have on policy and service planning. Evidence suggests that initial approach and personal connection greatly impacts on survey completion rates in all populations [35,36] which has implications for future survey planning, particularly as some appointments remain online.

Recent evidence has suggested that survey fatigue and nonparticipation, had increased as a result of the proliferation of online surveys during and following the COVID-19 pandemic [37]. Whilst the pandemic clearly impacted upon research in terms of recruitment and data collection, evidence suggests that the effects reached far wider. The pandemic exacerbated inequalities in health and access to health care, particularly amongst those living in economic hardship [38] and for Black African individuals and those of other minority ethnic groups [39], and the HIV population consists of a high proportion of these groups.

Strengths of PV2022 include the large sample size, the inclusion of a broadly representative sample of 1 in 20 people with HIV in England, Scotland and Wales (as evidenced by comparison with the national HARS database) and the linkage of participant self-reported questionnaire data to HIV clinical data from HARS. With increasing interest in patient experience and outcome measures, other clinical conditions could use a similar survey method to survey their patient group. PV2022 also has some limitations: as the study was only available to people with HIV in care, it did not represent those people with HIV who were not receiving care in 2022, whose mental and physical health, and experiences of health care, may be different and valuable for informing and improving HIV specialist services. Similarly, nonresponders to participate in the study may differ from responders with respect to health, lifestyle, and other factors. Further efforts to reach these groups and identify their needs are crucial. In addition, the sampling frame for the initial recruitment method was based on those attending care in 2020, and therefore our data are unable to represent those who have been newly diagnosed in the interim. Future work by the PV team will examine the recruitment challenges to PV2022 and will involve participant and public involvement groups and clinic staff. This will inform future rounds of the Positive Voices survey and optimize response rates.

PV2022 formed part of the formative work of the NIHR funded NICHE program of research led by UCL. NICHE aims to improve the mental and physical well-being of people with HIV by designing and evaluating a targeted psychosocial intervention as part of routine HIV care [29]. Findings from PV2022 informed the development of the intervention for the “Psycho-Social Intervention for People With HIV – Evidence From a Randomised Evaluation” (SPHERE) trial, which commenced in United Kingdom HIV clinics in August 2024. Results from Positive Voices 2022 have been published in a government report [30] that has directly informed the HIV action plan for England which aims to end stigma and inform interventions to improve patient-centered care and the provision of HIV clinical and support services. Further analysis of the data will be presented at HIV conferences, published in peer-reviewed journals and disseminated through the extensive NICHE PPI network.

## Appendix

Multimedia Appendix 1 Full list of participating clinics and clinic teams.

Multimedia Appendix 2 Table of Positive Voices 2022 (PV2022) Response rates* by recruitment mode, gender, ethnicity, and age among participants of PV2022 linked to HARS* (N=4622).

## Abbreviations

ART: antiretroviral therapy
AUDIT-C: Alcohol Use Disorders Identification Test-Consumption
GAD-7: Generalized Anxiety Disorder questionnaire-7
GP: general practice
GPPAQ: General Practice Physical Activity Questionnaire
HARS: HIV and AIDS reporting system
NICHE: A Person-Centered Needs Informed Model of Care for People with HIV
NIHR: National Institute for Health Research
PHQ-9: Patient Health Questionnaire-9
PV: Positive Voices
PV2017: Positive Voices 2017
PV2022: Positive Voices PV2022
PO-PROM: Positive Outcomes Patient Reported Outcome Measure
RS14: 14-item Resilience Scale
SPHERE: Psycho-Social Intervention for People With HIV–Evidence From a Randomised Evaluation
UKHSA: UK Health Security Agency
U=U: Undetectable equals Untransmittable
UCL: University College London
UNC: University of North Carolina

## Data Availability

The data sets generated during this study are not publicly available due to ongoing analysis and dissemination plans by the NICHE and UKHSA teams. However, data may be available from the corresponding author on reasonable request.
