# Correction: Methodology for the Positive Voices 2022 Survey of People With HIV Accessing Care in England, Wales, and Scotland: Cross-Sectional Questionnaire Study

**DOI:** 10.2196/72512

**Published:** 2025-02-27

**Authors:** Janey Sewell, Carole Kelly, Adamma Aghaizu, Hannah Kitt, Annegret Pelchen-Matthews, Veronique Martin, Amal Farah, Colette Smith, Alison Brown, Clare Humphreys, Alex Sparrowhawk, Valerie Delpech, Alison Rodger, Fiona Lampe, Meaghan Kall

**Affiliations:** 1 Department for Infection and Population Health University College London London United Kingdom; 2 UK Health Security Agency London United Kingdom; 3 George House Trust Manchester United Kingdom; 4 University College London London United Kingdom; 5 Royal Free London NHS Foundation Trust London United Kingdom

The paper “Methodology for the Positive Voices 2022 Survey of People With HIV Accessing Care in England, Wales, and Scotland: Cross-Sectional Questionnaire Study” published in JMIR Res Protoc 2025;14:e58531) had a few errors. Hence, the following edits have been made (changes italicized).

In the **Abstract,** the following subsections had a few sentences modified, as follows:

- Objectives: “This paper aimed to describe the methodology, recruitment strategies, and *key demographic* features of participants…”
- Methods: “…At the start of 2023, due to under-recruitment mainly due to the impact of the monkeypox (Mpox) outbreak, a separate sequential recruitment strategy was initiated in *14 of the largest clinics* to increase participant numbers.”
- Results: “…The median age of participants was 52 years, 3428 of participants were men, 2991 were *White*, and 1121 were *Black*.”

In the **Introduction** section, the following paragraphs have been revised, as follows:

- “High levels of perceived and internalized stigma associated with *HIV status are experienced* by people with HIV, impacting their mental health…
- PV is a cross-sectional *questionnaire study of people with HIV who receive HIV specialist care in England, Wales, and Scotland, which is carried out every 3-5 years…*
- Our paper aims to describe the methodology and study design of the second round of the *study: Positive Voices 2022 (PV2022).*”

In **Methods** section, the first column of ***Table 1*** has been edited to improve readability and clarity.

The following incorrect values were updated in ***Table 2***:

- The number of Positive Voices 2022 participants was changed from *4620* to *4622.*
- “Age group (years)” category: The percentage of participants in the 55-64 subcategory was changed from *30.2* to *30.3*.
- “Gender” category: The number and percentage of participants in the “Prefer not to say *and unknown*” subcategory was changed from *34 (0.7)* to *36 (0.8)*.
- “Ethnic group” category: The number and percentage of participants in the “Other (including prefer not to say and unknown)” subcategory was changed from *176 (3.8)* to *178 (3.9).*
- “Year of diagnosis” category: The number of participants in the “2019-2023” subcategory was changed from *213* to *214.*
- “Year of diagnosis” category: The number of participants in the “2014-2018” subcategory was changed from *800* to *801.*
- Footnote “a” was assigned to the “Age group (years)” category and was updated from *Missing sociodemographic information for 2 participants* to *Age was missing for 2 participants.*

A few abbreviations have been introduced for better readability. These have also been added to the abbreviations list at the end of the article.

A few additional changes have been listed below:

- Under Study Design:
  “The questionnaire data were linked to clinical data on antiretroviral therapy (ART), HIV viral load, and CD4 *lymphocyte* count from the HIV and AIDS reporting system (HARS)…”

- Under Setting:
  “178 HIV clinics in the country, returned an expression of interest, *98* % of whom were in England (99/101)…”

- Under Sample Size:
  “HARS was used as a sampling frame to *provide a representative,* random sample of people with *HIV within each participating clinic, who* could be approached to participate in the PV2022 survey. HARS is a surveillance database held at the UKHSA that consists of pseudonymized data on the demographic, clinical, and *treatment characteristics* of people with HIV that is reported each quarter year by all HIV service providers in England…
  …Therefore, considering this, a sample list of 17,121 patients was created *with the assumption* that approximately 14,400 would be recruitable. …the PV2022 response rate was expected to be between 30% and 50%, resulting in 4320 *to* 7200 participants being recruited. … ±1.4% and ±1.1% *(with 95% confidence)* for the response rates of 30% and 50%, respectively.”

- Under Study Management:
  “PV2022 was a collaboration between the University College London (UCL) NICHE *team, the UKHSA HIV national surveillance team, and a NICHE Patient and Public Involvement representative. “A Person-Centered Needs Informed Model of Care for People with HIV” (NICHE)* is a National Institute for Health Research (NIHR) funded program...”

- Under Questionnaire Development:
  “…These included demographic and socioeconomic factors, HIV related factors, comorbidities, met and unmet health, social and welfare *service* needs, quality of life measured by EQ-5D-5L, *height and weight*, smoking and alcohol status *by the Alcohol Use Disorders Identification Test- Consumption (AUDIT-C)*, recreational drug use and general practice (GP), and HIV clinic satisfaction, enabling assessment of trends over time in these factors…
  …New items included in the PV2022 questionnaire included… a modified version of the *Duke-UNC (University of North Carolina)* Functional Social Support Questionnaire…
  Questions on stigma and discrimination were expanded to take account of internalized *and other* stigma and included an adapted validated stigma scale [27]. In addition, participants *who* completed the study online in PV2022 were able to opt-in to complete “Positive Outcomes,” an HIV Patient Reported Outcome Measure (PO-PROM), a tool designed for use in clinical settings to assess *the needs and concerns of* people with HIV…”

- Under Study Documents:
  “...a password-protected study log that listed each clinic’s sample list of *randomly selected* participants. ... and unique identifier displayed on the outside, as well as the patient information leaflet, *questionnaire*, a freepost...”

- Under Linkage:
  “Data on ART *use*, the most recent CD4 lymphocyte count,...”

- Under Ethical Considerations:
  “All participants received a digital gift voucher for 5 GBP (*equivalent to* US $6.50)...”

In the **Results** section, the following sentences have been edited:

- Under Response Rate:
  “...the original preselected participant recruitment strategy (*49*%, Figure 1). Of the 930 sequentially recruited participants, 906 were able to be matched to HARS: 222 (*25*%) of these had initially been preselected to take part in the survey.
  Men had a higher response rate (60%) (3428 completed out of 5715 accepted or declined) compared with women (1124/2580, 44%) and *White people* had a higher response rate (2991/4724, 63%) compared with *Black African people* (983/2380, 41%) *or those of* other ethnicities (648/1158, 56%).”

- Under Age, Gender, *and Ethnic* Distribution of PV2022 Participants:
  “The median age of PV2022 participants (n=4620) was *52 (IQR 43-60) years, and about* 3 quarters of participants had been diagnosed with HIV more than 10 years ago (Table 2). Nearly a quarter of participants were women (24%) and over a fifth (21%) of participants were *Black African people* (Table 2).
  A total of *67* PV2022 participants identified as transgender or gender diverse...
  Compared with the national population of people accessing HIV care, *Black African individuals were underrepresented in the PV2022 sample (10% lower)*, and *White* participants were overrepresented (13% higher; Figure 2)...
*Participants from the sequential recruitment route were younger compared to the preselected random recruitment strategy (median age 49 (IQR 39-57) years versus 53 (IQR 45-60) years, for sequential versus random recruitment respectively), overrepresented men (85% vs 72%) and White people (67% vs 64%) and underrepresented Black African people (14% vs 23%).*
  A higher proportion of *participants who completed the online questionnaire were White compared to participants who completed the paper questionnaire (67% online vs 63% paper*; Figure 1 and Multimedia Appendix 2).”

In the **Discussion** section, the following edits were made:

- Under Principal Findings:
  “…The pandemic exacerbated inequalities in health and access to health care, particularly amongst those living in economic hardship [38] and for *Black African individuals and those of other* minority ethnic groups [39], and the HIV population consists of a high proportion of these groups.
  Strengths of PV2022 include the large sample size, the inclusion of a broadly representative sample of 1 in 20 people with HIV in *England, Scotland and Wales* (as evidenced by comparison with the national HARS database) and the linkage of participant self-reported questionnaire data to HIV clinical data from HARS.
  ..., whose mental and physical health, and experiences of *health* care, may be different and valuable for informing and improving HIV specialist services.
  Findings from PV2022 informed the development of the intervention for the “Psycho-Social Intervention for People With HIV–Evidence From a Randomised Evaluation” *(SPHERE)* trial, which commenced in United Kingdom HIV clinics in August 2024.”

The correction will appear in the online version of the paper on the JMIR Publications website on February 27, 2025, together with the publication of this correction notice. Because this was made after submission to PubMed, PubMed Central, and other full-text repositories, the corrected article has also been resubmitted to those repositories.

