# Supplementary material for: Methodology for the Positive Voices 2022 Survey of People With HIV Accessing Care in England, Wales, and Scotland: Cross-Sectional Questionnaire Study
Source: JMIR Res Protoc. 2025 Jan 10;14:e58531. doi: 10.2196/58531 (PMC11759904; doi:10.2196/58531)
Supplement: Multimedia Appendix 1 [file resprot_v14i1e58531_app1.docx]

**Appendix 1**: Full list of participating clinics and clinic teams

10 Hammersmith Broadway Clinic, London (Ann Sullivan, Rachel Jones, Mohammed Hassan, Serge Miodragovic)

56 Dean Street, London (Ann Sullivan, Victoria Tittle, Mohammed Hassan, Serge Miodragovic)

Abbey View Clinic, iCaSH Suffolk, Bury St Edmunds (Sarah Edwards)

Alexis Clinic, Alexis Clinic, London (Melanie Rosenvinge, Allison Mascagni, Rosa Harrington, Claudia Adade)

Axess Clinic, Bath Street Health and Wellbeing Centre, Warrington (Emily Clarke, Sandra Mason)

Axess Clinic, Eagle Bridge Health and Well Being Centre, Crewe (Emily Clarke, Elaine Priest)

Axess Clinic, Halton General Hospital (Emily Clarke, Sandra Mason)

Axess Clinic, Macclesfield Hospital (Emily Clarke, Elaine Priest)

Axess Clinic, Royal Liverpool University Hospital (Emily Clarke, Melissa Martin)

Barking Community Hospital Sexual Health Clinic, London (Athavan Umaipalan, Julie Field)

BBV Clinic, Milton Keynes University Hospital (Clare Woodward, Felicity Williams)

Beckenham Beacon Sexual Health, Kings College Hospital, London (Liz Hamlyn, Lucy Campbell)

Birmingham Heartlands HIV Service (Steve Taylor, Gerry Gilleran, Satwant Kaur)

Branston Clinic, Burton-on-Trent (Cathy Ormiston, Laura Wilson-Powell, Kate Saunders)

Breydon Clinic, iCaSH Norfolk, Great Yarmouth (Meena Gupta, Julia Ball)

Bristol HIV Service, Southmead Hospital (Mark Gompels, Louise Jennings, Malgorzata Slowinska)

Brookside Clinic, Aylesbury (Angela Bailey, Sandra Rushwaya)

Brotherton Wing Clinic, Leeds General Infirmary (Sarah Schoeman, Tadas Mazeika)

Buryfields Sexual Health Clinic, Surrey Sexual Health Service, Guildford (Shalini Andrews, Laura Noonan)

Caldecot Centre, Kings College Hospital, London (Liz Hamlyn, Lucy Campbell)

Cardiff Royal Infirmary (Darren Cousins, Catherine Oliver)

Chalmers Sexual Health Centre, Edinburgh (Daniel Clutterbuck, Connor Dalby, Amy Shepherd)

Chesterfield ISHS (Anura Piyadigamage, John Martin)

Chichester Sexual Health, St Richard’s Hospital (Judith Zhou, Barbara Hayman, Emma Rutland)

Churchill Hospital, Oxfordshire Sexual Health Service (Paola Cicconi, Charlie Wells)

Clinic 1a, Addenbrooke’s Hospital, Cambridge (Fiona Wilson)

Clinic 6, The Oaktree Centre, Huntingdon (Claudia Krause, Su Jenkins)

Clover Street Clinic, Chatham (Anitha Vidhyadharan, Samantha Harwood)

Cobridge Community Health Centre, Stoke-on-Trent (Lisa Goodall, Alison Bridgwood, Laura Wilson-Powell)

Coelho Clinic, Chelmsford (Suzanne Francis, Kirsty Mynard, Mandy Austin)

Crawley Sexual Health, Crawley Hospital (Judith Zhou, Farai Mukazi, Chloe Hoskins)

Croydon Sexual Health Centre, Croydon University Hospital, London (Ian Cormack)

Devon Sexual Health, Barnstaple (Jonathan Shaw, Amanda Smith)

Devon Sexual Health, Torbay (Nadia Khatib, Julie Walsh)

Dewsbury Health Centre (Sarah Schoeman, Tadas Mazeika)

East Kent HIV Service, Folkestone Health Centre (Anitha Vidhyadharan, Brenda Hollier)

East Sussex Sexual Health, Eastbourne (Martin Jones, Penny Boxall)

Florence Nightingale Community Hospital, Derby (Ade Apoola, Catherine Gatford)

Fountains Sexual Health Clinic, Chester (John Evans-Jones, Jennifer Harrison)

Grahame Hayton Unit, Royal London Hospital, London (Nashaba Matin, Moses Shongwe)

Greenway Centre, Newham General Hospital, London (Nashaba Matin, Moses Shongwe)

Harrogate Sexual Health Centre (Ian Fairley)

Hastings Clinic, Station Plaza, East Sussex Sexual Health (Martin Jones, Zoe Cuthbertson, Penny Boxall)

Hathersage Centre, Manchester Centre for Sexual Health (Chitra Babu, Denise Donahue)

Ian Charleson Day Centre, Royal Free Hospital, London (Fiona Burns, Katie Spears, Thomas Fernandez)

iCaSH Peterborough (Graham McKinnon, Rachael Bridgman)

Kobler Clinic, Chelsea and Westminster Hospital, London (Ann Sullivan, James Hardie, Mohammed Hassan, Serge Miodragovic)

Lawson Unit HIV Clinic, University Hospitals Sussex NHS Foundation Trust, Brighton (Amanda Clarke, Lisa Barbour, Carole Cable)

Luton Sexual Health (Mohanarathi Kawsar, Memory Kakowa)

Mortimer Market Clinic, London (Richard Gilson, Gosala Gopalakrishnan, Abigail Severn)

Newington Road Clinic, Ramsgate (Anitha Vidhyadharan, Kate Castro-Sanchez)

North Manchester General Hospital (Andrew Ustainowski, Fahd Niaz)

Northampton General Hospital (Sophie Herbert, Helen Reboul)

Nottingham Sexual Health Service, Nottingham City Hospital (Ashini Fox, Sarah Chadwick)

Oak Street Clinic, iCaSH Norfolk, Norwich (Nelson David, Megan Khan)

Open Clinic, Bishton Court, Telford (Andrea Ng, Julia Rogers, Katie Saunders)

Open Clinic, Sexual Health Services - Shropshire, Shrewsbury (Andrea Ng, Julia Rogers, Katie Saunders)

Open Clinic, Stafford (Cathy Ormiston, Amandeep Gill, Laura Wilson-Powell, Katie Saunders)

Portsmouth Sexual Health Service (Alison Blume, Natalie Parker)

Queen Elizabeth Hospital, Birmingham (Jonathan Ross, Sindiso Masuka)

Rosehill Clinic, St Helier Hospital, London (Olubanke Davies, Analyn Alipustain, Maheshraj Radhakrishnan)

Rotherham General Hospital (Nadi Gupta, Nicola Williams)

Salisbury District Hospital (Helen Iveson)

Scarborough Sexual Health Centre, The Mulberry Unit (Ian Fairley)

Sexual Health at Wycombe (Angela Bailey, Sandra Rushwaya)

Sexual Health Calderdale, Broad Street Plaza, Halifax (Emma Street, Andrew Sealy)

Sexual Health Clinic, Monkgate Health Centre, York (Ian Fairley, Tom Yucebiyik)

Sexual Health Dorset, Bournemouth (Elbushra Herieka, Kevin Turner)

Sexual Health Service, Isle of Wight (Alison Blume, Felicity Young)

Sexual Health Sheffield, Royal Hallamshire Hospital, Sheffield (Karen Rogstad, Jessica Mcneill, Gareth Stephens)

SHiP, Derriford Hospital, Plymouth (Zoe Warwick, Angela Robinson, Elaine Freeman)

Sir Ludwig Guttman Centre, Stratford, London (Nashaba Matin, Moses Shongwe)

Southend Hospital (Laura Hilton, Donna Stookes)

Spectrum Community Health, Wakefield (Sarah Schoeman,Tadas Mazeika)

St Helens Hospital Sexual Health (genitourinary medicine (GUM)) Service (Elizabeth Okecha)

St Lukes Hospital, Bradford (Nicola Fearnley, Jackie Todd, Sue Kimachia)

Stevenage Clinic (Ann Sullivan, Sarah Edwards, Mohammed Hassan)

Summers Unit, Kettering Hospital (Sophie Herbert, Helen Reboul)

Swindon Sexual Health Department, The Great Western Hospital (Jessica Daniel, Mary-Jane Harding)

The Centre, Sidwell Street, Exeter (Jonathan Shaw, Abbey Eboigbe, Ashley Hanson)

The Courtyard Clinic St George’s Hospital, London (Liz Hamlyn, Katie Toler)

The Florey Sexual Health Services, Royal Berkshire Hospital, Reading (Fabian Chen, Emma Wainwright, Felix Kpodo)

The Garden Clinic, Upton Hospital, Slough (Nisha Pal, Clare Megson)

The Gate Clinic, Canterbury (Anitha Vidhyadharan, Matt Waller)

The James Cook University Hospital, Middlesborough (David Chadwick, Jessica Roberts)

The Jonathan Mann Clinic, Homerton Hospital, London (Iain Reeves, Tracey Fong)

The Orwell Clinic, iCaSH Suffolk, Ipswich (Raouf Moussa, Melissa Milsom)

The Portland Clinic, Huddersfield Royal Infirmary (Emma Street, Mike Ward)

The Riverside Clinic, Riverside Health Centre, Bath (Lucy Twigger, Charlotte Swift)

The Royal South Hants Hospital, Southampton (Raj Patel, Jane Whitehead)

The Starling Clinic, Musgrove Park Hospital, Taunton (Sathish Thomas William, Jane Holder)

The Trafalgar Clinic, Queen Elizabeth Hospital, Greenwich, London (Stephen Kegg, Rosa Harrington, Allison Mascagni, Claudia Adade)

The Wolverton Centre for Sexual Health, Kingston Hospital, London (Lewis Haddow, Jessica Osorio)

Twickenham House, West Middlesex Hospital, London (Ann Sullivan, Marie-Louise Svensson)

Vancouver House, iCaSH Norfolk, King’s Lynn (Sandra Underwood, Helen Pollitt)

Vicarage Lane Clinic, Ashford (Anitha Vidhyadharan, Brenda Hollier)

Watford Clinic (Ann Sullivan, Samantha Hill)

Weymouth Community Hospital, Sexual Health Dorset, Weymouth (Sara Scofield, Jenny Murira)

Wharfside Clinic, St Mary’s Hospital, London (Nicola Mackie, Sophia Taylor, Romina Tajik)

Withington Community Hospital, Manchester (Orla McQuillan, Denise Donahue)

Worthing Sexual Health (Judith Zhou, Rebecca Murdock, Elaine Banks)
