# Supplementary material for: Methodology for the Positive Voices 2022 Survey of People With HIV Accessing Care in England, Wales, and Scotland: Cross-Sectional Questionnaire Study
Source: JMIR Res Protoc. 2025 Jan 10;14:e58531. doi: 10.2196/58531 (PMC11759904; doi:10.2196/58531)
Supplement: Multimedia Appendix 2 [file resprot_v14i1e58531_app2.docx]

Appendix 2. Table of PV2022 Response rates* by recruitment mode, gender, ethnicity, and age among participants of PV2022 linked to HARS* (N=4622).

|  |  | approached | Completed | | | Response rate |
| --- | --- | --- | --- | --- | --- | --- |
|  |  |  | Paper | Online | Total |  |
|  | Overall | 9,184 | 2,829 | 1,793 | 4,622 | 50% |
| Recruitment strategy | Random | 7,517 | 1899 | 1793 | 3,692 | 49% |
|  | Sequential | 1667 | 930 | 0 | 930 | 56% |
| Gender | Men (including trans men) | 5,715 | 2089 | 1339 | 3428 | 60% |
|  | Women (incuding trans women) | 2,580 | 706 | 418 | 1124 | 44% |
| Ethnic group | White | 4,724 | 1785 | 1206 | 2991 | 63% |
|  | Black African | 2,380 | 610 | 373 | 983 | 41% |
|  | Other ethnicities | 1,158 | 434 | 214 | 648 | 56% |
| Age group | Aged 18 to 34 years | 841 | 207 | 162 | 369 | 44% |
|  | Aged 35 to 44 years | 1,863 | 500 | 385 | 885 | 48% |
|  | Aged 45 to 54 years | 2,705 | 813 | 562 | 1375 | 51% |
|  | Aged 55 to 64 years | 2,115 | 892 | 505 | 1397 | 66% |
|  | Aged 65 years and over | 851 | 413 | 179 | 592 | 70% |
